# Research on differential privacy protection method based on user tendency

**DOI:** 10.1371/journal.pone.0288823

**Published:** 2023-10-26

**Authors:** Zhaowei Hu

**Affiliations:** 1 School of Computer Science and Artificial Intelligence, Changzhou University, Changzhou, Jiangsu, China; 2 College of Computer, Jilin Normal University, Siping, Jilin, China; Jeonbuk National University, REPUBLIC OF KOREA

## Abstract

It is a new attack model to mine user’s activity rule from user’s massive data. In order to solve the privacy leakage problem caused by user tendency in current privacy preserving methods, an extended differential privacy preserving method based on user’s tendency is proposed in the paper. By constructing a Markov chain, and using the Markov decision process, it equivalently expresses user’s tendency as measurable state transition probability, which can transform qualitative descriptions of user’s tendency into a quantitative representation to achieve an accurate measurement of the user tendency. An extended (P,ε)-differential privacy protection method is proposed in the work, by introducing a privacy model parameter R, it combines the quantified user’s propensity probability with a differential privacy budget parameter, and it can dynamically add different noise amounts according to the user’s tendency, so as to achieve the purpose of protecting the user’s propensity privacy information and improve data availability. Finally, the feasibility and effectiveness of the proposed method was verified by experiments.

## 1. Introduction

With the continuous development of the Internet of Things and the Data Mining Technology, it is a new attack model to mine user’s activity patterns from a large amount of user data [1–4]. Based on the user’s historical trajectory data, the attacker first models the activity patterns of the target user, and he makes a specific quantitative description of it. Then, according to the constructed model, the attacker recovers and reconstructs the user’s trajectory to speculate the sensitive information hidden by the user, to predict the possibility of the user’s access to a certain geographical location, and to predict the user’s trajectory and path.

User’s movement behavior is often closely related to the time attribute, and the user’s activity patterns can reflect his tendency, which is very important for the attacker to analyze the user’s behavior attribute [5]. The more balanced the probability of users visiting different locations is, the less obvious the behavioral tendency is shown, and the smaller the risk of privacy information is disclosed. The more balanced the probability of users accessing different locations is, the less obvious their behavioral tendency is. The less tendentious a user is shown, the less the risk of privacy information is leaked. Therefore, only the tendency of users is protected in the data set, it can effectively protect the user’s privacy information and prevent sensitive information from being leaked.

In the paper, it aims to protect the tendency of mobile users, it is based on the state transitions in Markov models, a Markov chain model is constructed to quantify the characteristics of the probability transformation between adjacent location points. The probability characteristic and privacy budget parameter are associated to dynamically add noise and protect user tendency to prevent user’s private information from being leaked. The main work done in the paper is as follows.

First, an extended differential privacy protection method is proposed to protect a user’s tendency based on the Markov decision process. By analyzing the user’s historical trajectory data and calculating the probability of the user’s access to specific location points, the user’s tendency probability is converted into the state transition probability of a Markov chain. It uses the state transition probability of the location point to quantify the user’s tendency, and it protects the trajectory whose tendency is higher than the threshold to prevent the leakage of private information.

Second, in the basis of traditional ε-differential privacy protection method, by constructing a Markov chain and introducing privacy model parameter, user’s personal tendency is quantified and measured. It combines user tendency probability and differential privacy budget, which can dynamically add different and appropriate noise according to control user visit tendency, to protect user sensitive information and improve data availability.

Third, the feasibility and effectiveness of the proposed method are demonstrated by comparing with the other two privacy protection methods for analysis. The results show that the proposed method can provide efficient privacy protection and the data availability.

The paper is organized as follows: Section 2 discusses related work, and section 3 presents research content and makes a statement about the research question, section 4 systematically introduces research methods and introduces the methods of measuring the effectiveness of privacy protection. An analysis and discussion of the experiments are provided in section 5. Section 6 concludes the paper.

## 2. Related work

A user’s location and trajectory data implies the user’s activity law and personal tendency, according to the tendencies, an attacker can deduce the user’s sensitive information from the anonymous data and predict the possibility of the user to visit certain geographical locations. The activity rule of user himself and similar groups affect the next access location of user with different probabilities. The attack based on user’s tendency depends on the characterization of specific attack targets by the model, it uses the privacy model of the user’s location trajectory as the user profile to re-identify and de-anonymize the user [6–10]. Ashbrook firstly applied the Markov model to the analysis of geographical location information, it built a Markov model for users based on their temporal transfer characteristics of locations and predicted the next location of users based on the user movement Markov model [11].

Alvarez combined the user’s trajectory information with the local road network information to accurately predict the user’s current travel destination [12]. Gambs obtained the POI of user trajectory after clustering based on location density and calculated the transition probability between POI with Mobility Markov Chain [13]. Pan updated the discrete time Markov chain model to the continuous time one [14], which can better simulate the state change process of user’s stay and transfer. However, when the user’s behavior trajectory changes, it takes a long time to complete the model update.

Wang believed that user’s mobile behaviors is often closely related to time, and the time attribute contained was very significant for analyzing user’s mobile behaviors [15]. In practical applications, Researcher found that user’s mobile behavior was centrality, that is, users were centered around several geographic locations. Gonzalez studied the movement pattern of users through mobile phone data, he found that people regularly return to a small number of previously visited locations, and the movement pattern can be modeled as a random process centered on a fixed point [16]. Song demonstrated that 93% of human movements are highly regular, and the user spends about 70% of his time at the location which he visits most frequently [17].

Sadilek used dynamic Bayesian networks to predict the location by using friends’ historical trajectory and situational information [18]. On the basis of considering the problem of data sparseness, Xue divided the trajectory into several sub- trajectories and used them to generate R-order reachable transition matrix to expand the prediction space, it predicted all positions through Bayes and returned the extracted top-N positions to achieve more accurate user position prediction [19]. Huo used the Bayesian model as a hidden location reasoning attack model to obtain the behavior pattern and POI preference from the historical data of user, and she used the behavior patterns of most users to infer the likelihood that a user will visit a POI [20].

In order to analyze the influence of user’s activity rules on his mobile data and protect his privacy information, Qiu proposed a semi-supervised learning attack model [21]. By analyzing user’s mobile characteristics and life rules, it can identify important locations such as residence and workplace from his encrypted mobile data, and its accuracy can reach more than 98%. In order to solve the privacy leakage problem caused by user activity rules in mobile data sets, Tu proposed a method to recover user trajectories from aggregated mobile data [22]. Based on user’s personal characteristics or activity rules, 73–91% of personal trajectories can be recovered from anonymous mobility data without any prior knowledge. In order to hide the sensitive labels and the user’s activity rule which were contained in the user trajectory data, Yao proposed a comprehensive orbital protection trajectory publishing algorithm [23]. It determined the hot spots and outliers by density clustering, and it obfuscated the precise position by generalization. It captured the relationship between sensitive labels and trajectory points in all records and added Laplacian noise for differential privacy protection. In order to protect sensitive information such as user activity rules contained in the trajectory data, Buchholz proposed a protected trajectory reconstruction model based on deep learning [24], which used the difference of noise to reconstruct the original track, so as to reduce the Euclianian distance and Hausdorff distance between the published trajectory and the original one to protect the privacy information. Su proposed a POI recommendation algorithm which integrated social relationship and geographical influence to solve the problem of sparse data in users’ access points of interest [25]. It considered the privacy protection of activity rules in user trajectory data, and adopted graph convolutional neural network to explicitly learn the cooperative relationship between user and user, POI and POI, user and POI, so as to alleviate the problem of data sparsity and protect user’s privacy information.

However, the current research on the personal characteristics and activity rules of user’s mobile data which mainly focuses on the analysis, modeling and prediction of the regularity of user’s activities, it can identify the user’s activity rules and behavior attributes which were implied in the location trajectory data. They do not systematically analyze and mine the user’s activity rules, and summarize the user’s tendencies, and then to protect the tendencies. The analysis of user’s activity data sets show that user’s daily location and trajectory data show obvious tendencies, 53% of the check-in locations in Brightkite and 31% of the check-in locations in Gowalla are previously visited by users. The reflected tendency is very important to analyze the user’s activity rules in mobile data. It is not only necessary to protect the user’s location, trajectory and identity privacy, but also to protect the tendency of user, so as to effectively prevent the disclosure of user’s privacy information. At present, the research on differential privacy protection method has made a great progress, how to use differential privacy protection method to protect user’s tendency is the focus of the paper.

In order to reduce data distortion and improve data utilization, Comas proposed an improved differential privacy solution that could provide the same privacy guarantee as standard differential privacy [26]. They believed that the standard formalization of differential privacy was stricter than the intuitive privacy guarantee. In practical applications, the indistinguishability between a data set and its neighboring data sets were sufficient. Zhao proposed a privacy protection method based on differential privacy clustering; it added Laplacian noise to the trajectory position count in the cluster to resist continuous query attacks [27]. The noise cluster center was obtained via the noise location data and the noise location count. The noise cluster center was used to prevent excessive noise from affecting the clustering effect, and to ensure the availability of data in clustering analysis. Cao proposed a differential privacy protection method based on time correlation for continuous data release [28]. They designed an efficient algorithm that calculates a temporary privacy leakage and converted traditional differential privacy protection mechanisms into temporary privacy leakage mechanisms. These methods could effectively enhance the protection effect of user trajectory data and improve the availability of data, but they did not analyze and protect the personal tendency which was contained in user trajectory data.

Zhang proposed a differential privacy protection method based on probability mechanisms, which used a probability counting structure to count the number of users in various areas, and added noise extracted from the Laplace distribution to achieve disturbance. Additionally, a user could control the privacy level by adjusting the parameters of Laplace distribution [29]. To solve the problem of privacy protection in the release and use of mobile population perception data, Kim proposed a new type of cooperative game privacy protection model. According to personal preference, it provided effective payment solutions for each participating device to protect personal privacy information [30]. Gao solved the feedback Nash equilibrium solution through dynamic programming based on the MCS system, which could make users and platforms to maximize privacy requirements and data utility respectively, to solve the trade-off between privacy protection and data utility [31]. Cho believed that the user movement behavior pattern was consistent with the characteristics of Gaussian distribution, and the mixed Gaussian model could be used to model the user movement [32]. Analysis of the Brightkite dataset shows that if a user visits a location for the first time, there is a 53% probability that he will visit it again. Thus, it can be seen that the propensity of user’s activities can greatly reflect the behavior characteristics and life patterns, and it is very important to adopt the differential privacy method to protect user’s propensity privacy from disclosure.

To sum up, the current research analyzes, models and predicts the activity rules contained in mobile user trajectories, and builds a de-anonymization attack model based on user’s activity rules. However, they do not protect user’s tendency by combining attack models. With the continuous accumulation of data and the development of data mining, attacks will also increase based on user’s activity pattern and tendency. If privacy protection cannot be carried out according to user’s tendency, the attacker can predict the possibility of a user visiting certain sensitive locations based on the tendency revealed from a user’s historical data. Thus, the attacker can predict the starting point and ending point of his journey and an accurate path to mine his hidden sensitive information. In the paper, the traditional differential privacy protection model is extended to protect the user’s tendency, by constructing the transition state of Markov chain, it quantifies the user’s tendency as the state transition probability of Markov chain, and the user orientation probability is dynamically correlated with the privacy budget parameter of the differential privacy protection model. It can realize the dynamic addition of disturbing noise, so as to control the user’s access tendency, to protect his sensitive information and improve the data availability.

The differences between the done work of this paper and the previous work are as follows: In the first place, a trajectory privacy protection method based on user orientation is proposed to protect user’s tendency. By analyzing the historical trajectory data of user and calculating the probability of user’s accessing location points, it converts the user’s orientation probability to the state transition probability of Markov chain, and uses the state transition probability to quantify the user orientation. If a user’s preference is higher than the privacy protection threshold, it needs to be protected to prevent privacy information leakage. In the next place, a (P, ε)-differential privacy protection methods have been proposed to improve the data availability, by constructing a Markov Chain and introducing the privacy model parameters, the user’s propensity probability and differential privacy budget parameters are dynamically correlated, to dynamically add differential noise according to user preference probability, to control his access tendency and protect his sensitive information.

In the paper, the Markov model and the differential privacy protection model are combined to propose an extended differential privacy protection model based on user’s tendency, which is mainly based on the following three reasons: Firstly, a user’s movement trajectory is actually a collection of location points in chronological order, and the transition of location points has the property of Markov state transition, which can be regarded as a Markov decision process. Secondly, the user’s tendency reflects the transition probability of the user from one location point to another, which conforms to the Markov property and is suitable for processing with the state transition probability of the Markov model. Thirdly, the greater probability of a user visiting a specific location is, the more obvious its tendency is; which means that its sensitivity is higher, and the stronger privacy protection is required. Therefore, more noise needs to be added, which can be achieved by the differential privacy protection method.

## 3. Problem statement

### 3.1 User’s tendency attack model

With the in-depth development of data mining analysis, the new attacks based on user activity patterns and personal tendencies are becoming increasingly active. An attacker uses a specific model to quantitatively describe the activity of the target user and analyzes the regularity user’s access to certain physical locations, so as to obtain the user’s tendency and private information in the process of moving. In this way, the attacker can obtain the likelihood of user visiting a certain physical location, and even accurately predict his itinerary.

**Fig 1:**
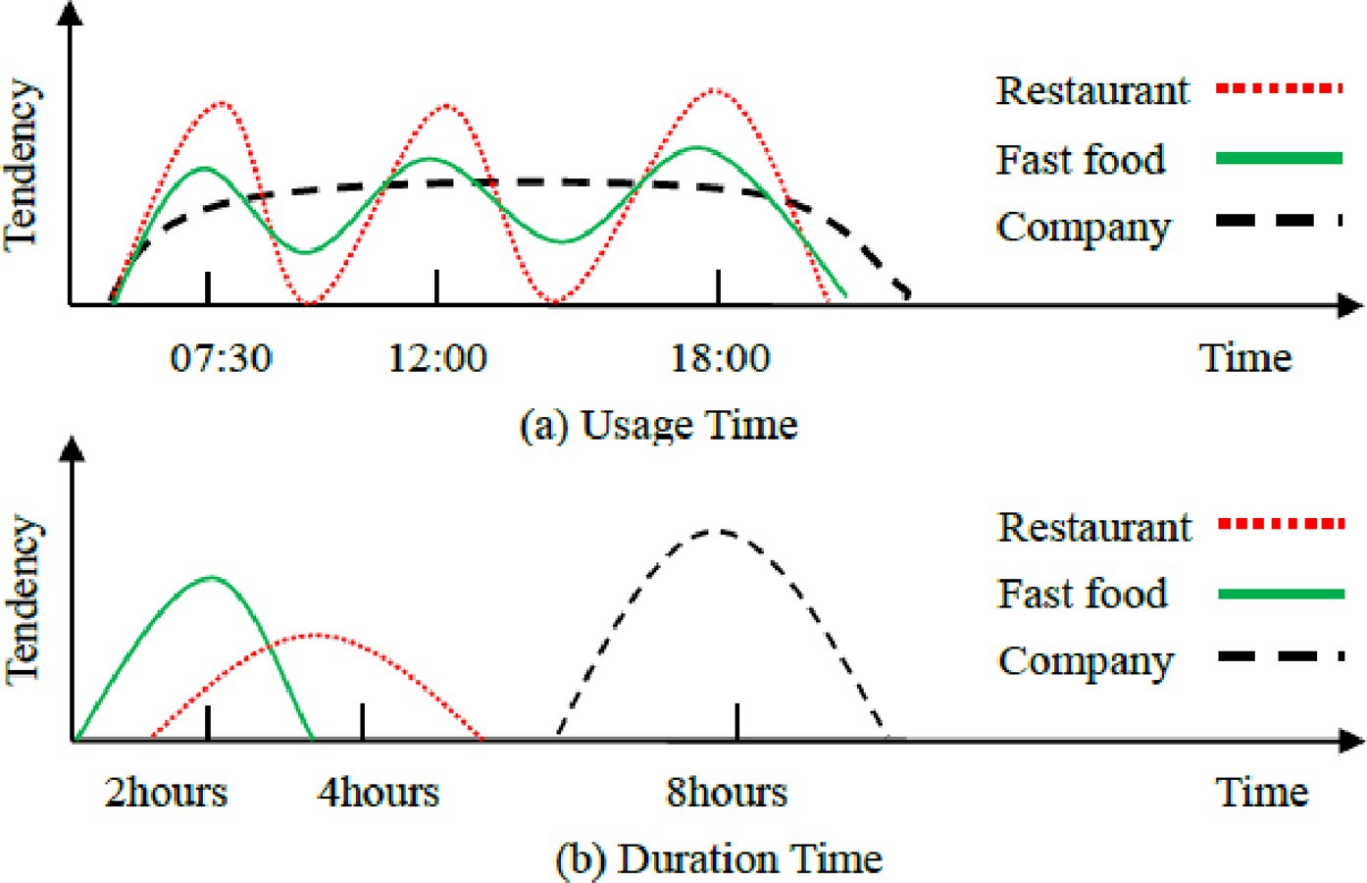
Schematic diagram of user tendency. (a) Usage Time. (b) Duration Time.

By analyzing the historical trajectory data of mobile user, the user’s movement regularity or tendency can be obtained. For example, people generally start from the residential area to the work area in the morning, from the work area to the commercial area in the afternoon, and finally from the commercial area to the residential area. If the service functions and geographical attributes of the places represented by the location points are different, the staying duration, and usage time of people are also different. As shown in Fig 1, people generally eat in restaurants at 07:30, 12:00 and 18:00, work at company from 7:30 to 18:00. The total time spent in fast food per day will not be more than 2 hours, while dining in restaurants will exceed 4 hours, but at company it will exceed 8 hours. The length of time that people visit different places and the transition of visiting places in different time periods are related to the user’s living habits, activity patterns and personal preferences, which reflects the user’s personal tendency.

The attacker collects the user’s historical trajectory data, which can analyze the real probability of the user visiting a location:

$$\text{P}=\left\{u_{p}^{t}\right\}$$ (1)

Where *u* represents the attacked user, attacked user A is represented as *u*_A_. *p* represents all the information sets of the user at a certain location, and *t* represents the time when the user *u* is at this location.

The attack based on the user’s tendency is that the attacker analyzes his activity pattern from his historical trajectory data, then it obtains the user’s tendency and predicts the possible future activities to obtain his private information. Assuming that at time *t*, the probability that the attacker can infer the location of the user is:

$$\text{P}=\left\{u_{p}^{t}|B\right\}$$ (2)

Where *B* represents the user tendency information obtained by the attacker, the probability is called the posterior probability, which is the user’s state transition probability and can be calculated from the user’s previous state. The whole model represents the probability that the attacker can infer the user’s location when he has obtained the above information.

The amount of prior information that the user is in a certain position at time *t* is $\sum_{p}P\left\{u_{p}^{t}\right\}\log P\left\{u_{p}^{t}\right\}$, then the amount of position information is exposed to the attacker is [33, 34]:

$$\text{M}=\sum_{p}\;P\left\{u_{p}^{t}\right\}\log P\left\{u_{p}^{t}\right\}-\sum_{p}\;\text{P}\left\{u_{p}^{t}|B\right\}\log\text{P}\left\{u_{p}^{t}|B\right\}$$ (3)

When M is higher than the set threshold, that is, when it reaches the propensity attack threshold, the attacker can analyze the user’s activity regular pattern and personal preference according to the prior probability of user access and the posterior probability calculated by statistics, and it can conduct attacks based on user tendency to obtain user’s private information. Therefore, the protection of a mobile user’s tendency is an important content of user privacy protection. When establishing a privacy protection model, the tendency probability *p* must be reduced to a certain threshold to ensure the validity of the model, so that an attacker cannot predict or speculate the user’s next state based on his inclination and current state, to conduct tendentious attacks and obtain his private information.

### 3.2 User tendency analysis based on Markov decision process

A trajectory is a sequence of location points that a mobile user visits in chronological order. The possibility of a user visiting a location is only related to the location visited at the previous moment, not the location visited before. This feature is consistent with the properties of state point changes in Markov models. Therefore, the position points on the user’s trajectory can be regarded as the state points in the Markov model, and the user’s tendency is analyzed by analyzing the regularity of the state transition in the Markov chain. Assuming that the states (position points) of the first *n* moments on the user’s trajectory are X_0_,X_1_,X_2_,…X_n_, the probability of the state *x* at the *n+1* moment can be obtained from the Markov chain.

***Definition 1*:** For a random sequence {X(s,t),t∈T,s∈S}, a time parameter set T = {t_n_|t_n_≥0,n∈N^*^}, the corresponding state space value is {n = 0,1,2,…}, for any t_n_∈T and the state i_n_ at time t_n_, then the probability that the state of sequence X is i_n+1_ at time t_n+1_ is:

$$\text{P}\left\{\text{X}\left({\text{t}}_{\text{n}}\text{+1}\right){\;\text{=i}}_{\text{n+1}}|\text{X}\left({\text{t}}_{\text{1}}\right){\;=\;\text{i}}_{\text{1}},\ldots ,\text{X}\left({\text{t}}_{\text{n}}\right){\;=\;\text{i}}_{\text{n}}\right\}$$

$$\text{=P}\left\{\text{X}\left({\text{t}}_{\text{n+1}}\right){\;=\;\text{i}}_{\text{n+1}}|\;\text{X}\left({\text{t}}_{\text{n}}\right){\;=\;\text{i}}_{\text{n}}\right\}$$ (4)

The sequence {X(t),t∈T} is called a Markov chain, i.e. given past states: X^(0)^, X^(1)^,X^(2)^, X^(3)^, X^(4)^,…,X^(n-1)^, and the current state X^(n)^, then the future state X^(n+1)^ is independent of the past state, and the conditional probability that it is only related to the current state can be expressed as:

$$\text{P}\left({\text{X}}_{\text{n+1}}{\text{=x}}_{\text{j}}{|\text{X}}_{\text{0}}{\text{=x}}_{\text{0}}{\text{,X}}_{\text{1}}{\text{=x}}_{\text{1}}{,\ldots ,\text{X}}_{\text{n}}{\text{=x}}_{\text{i}}\right)\;=\;\text{P}\left({\text{X}}_{\text{n+1}}{\text{=x|X}}_{\text{n}}{\text{=x}}_{\text{i}}\right){\text{=P}}_{\text{ij}}$$ (5)

A Markov chain is a stochastic process with Markov properties, it is characterized by the fact that the next adjacent state is only related to the current state, but not to the past state. In the Markov chain, the state of a random process can be converted to another after a certain period time, which is called the state transition probability. The value of the probability can reflect the regularity and preference of user behavior, that is, user orientation.

***Definition 2*:** Given a Markov chain {X(t),t∈T}, the one-step transition probability at time t_n_ is:

$${\text{p}}_{\text{ij}}\left(\text{n}\right)\text{=p}\left\{\text{X}\left({\text{t}}_{\text{n+1}}\right){\text{=j}}_{\text{n}}|\text{X}\left({\text{t}}_{\text{n}}\right){\text{=i}}_{\text{n}}\right\}$$ (6)

Where *p*_*ij*_ represents the probability that the sequence transitions from state *i* at time *t*_*n*_ to state *j* at time *t*_*n+1*_., i,j∈S, one-step transition probability is referred to as transition probability. The transition probability is satisfied with the following properties:

$$p_{ij}\geq 0,\sum_{i-0}^{\infty }p_{ij}=1,j=0,1,2,\ldots$$ (7)

It assumes a sequence with *n* states; the one-step transition probability from state *i* to state *j* is denoted as p_ij_, then the matrix composed of one-step transition probabilities at all time is called the probability transition matrix, which is expressed as:

$$P=\left[\begin{array}{cccc} p_{11} & p_{12} & \ldots & p_{1n}\\ p_{21} & p_{22} & \ldots & p_{2n}\\ \ldots & \ldots & \ldots & \ldots \\ p_{n1} & p_{n2} & \ldots & p_{nn} \end{array}\right]$$ (8)

**Example**. A Markov chain with three states is constructed and its state transition probability matrix *p* as follows:

1. When X^(n)^ = 0, then P(X^(n+1)^ = 0) = 0.1, P(X^(n+1)^ = 1) = 0.4, P(X^(n+1)^ = 2) = 0.5.
2. When X^(n)^ = 1, then P(X^(n+1)^ = 0) = 0.6, P(X^(n+1)^ = 1) = 0.2, P(X^(n+1)^ = 2) = 0.2.
3. When X^(n)^ = 2, then P(X^(n+1)^ = 0) = 0.3, P(X^(n+1)^ = 1) = 0.1, P(X^(n+1)^ = 2) = 0.6.

$$p=\begin{array}{c} 0\\ 1\\ 2 \end{array}\left[\begin{array}{ccc} 0.1 & 0.6 & 0.3\\ 0.4 & 0.2 & 0.1\\ 0.5 & 0.2 & 0.6 \end{array}\right]$$

The transition probability is the tendency of being in state *i* at time *n* and in state *j* after a certain time interval. According to the trajectory division method proposed in the literature [35, 36], when the Markov chain is modeled and the tendency analysis is performed, user’s trajectory data can be serialized and divided into sub-trajectories that only contain two adjacent points. Finally, these sub-trajectories are spliced into a complete trajectory. Tendency analysis is to evaluate the possibility of a direct state transition via the state transition matrix.

***Definition 3*:** The prior probability of user visiting a location point. The trajectory *T* is a state sequence composed of *n* position points *L*, where the *ith* position point is represented by *L*_*i*,_ and the value of state is represented by *l*_*i*_, then the probability of a user visiting it is represented as:

$$\text{P}\left({\text{L}}_{\text{i}}\text{=}l_{\text{i}}{|\text{L}}_{\text{i-1}}\text{=}l_{\text{i-1}}{\text{,L}}_{\text{i-2}}\text{=}l_{\text{i-2}}{,\ldots ,\text{L}}_{\text{1}}\text{=}l_{\text{1}}\right)\text{=P}\left({\text{L}}_{\text{i}}\text{=}l_{\text{i}}{|\text{L}}_{\text{i-1}}\text{=}l_{\text{i-1}}\right)$$ (9)

Therefore, the occurrence probability of any state is only related to the previous state, and it has nothing to do with other states. From the probability of the position point, the occurrence probability can determine the probability of trajectory T.

For any state L_i_, its prior probability is:

$$\text{P}\left(L_{i}\right)=\frac{num\left(l_{i}\right)}{\sum_{l_{j}\in \Sigma }count\left(l_{j}\right)}$$ (10)

Where num(*l*_*i*_) represents the number of occurrences of state *L*_*i*_, and *l*_*j*_∈∑ represents the sum number of all states.

The posterior probability is the transition probability from one position point to another, therefore it can be obtained through the state transition matrix. For any trajectory sequence T, the occurrence probability of each position point L is calculated separately, and the maximum value of all probabilities is regarded as the propensity probability of the trajectory sequence T.

***Definition 4*:** User’s tendency probability. The tendency probability is the spatial attribute contained in the user’s trajectory; it is the probability expression of a user’s regularity and willingness to visit a specific location and place. It is also the probability expression of a user’s personal preference. This can be quantitatively expressed as the state transition probability in the user’s trajectory, which is defined as:

$$\text{P}\left(\text{T}\right){\text{=P(L}}_{\text{n}}\text{=}l_{\text{n}}{|\text{L}}_{\text{n-1}}\text{=}l_{\text{n-1}}{\text{)P(L}}_{\text{n-1}}\text{=}l_{\text{n-1}}{|\text{L}}_{\text{n-2}}\text{=}l_{\text{n-2}}{)\cdots \text{P}(\text{L}}_{\text{1}}\text{=}l_{\text{1}}\text{)}$$

$$\text{=P(}l_{\text{n}}|l_{\text{n-1}}\text{)P(}l_{\text{n-1}}|l_{\text{n-2}})\cdots \text{P}\left({\text{s}}_{\text{1}}\right)$$

$${\text{=P(L}}_{\text{1}}\text{=}l_{\text{1}}\text{)}\prod_{\text{(i=2)}}^{\text{n}}P{\text{(L}}_{\text{i}}\text{=}l_{\text{i}}{|\text{L}}_{\text{i-1}}\text{=}l_{\text{i-1}}\text{)}$$

$$=P(l_{1})\prod_{\left(i=2\right)}^{n}P(l_{i}|l_{i-1})$$ (11)

Where P(*l*_1_) represents the probability that the user accesses the first position point of trajectory T, and P(*l*_i_) represents the probability that the user accesses the i-th position point of the trajectory T, which is the real probability that the state *l*_*i*_ occurs, it is the prior probability. P(*l*_i_|*l*_i-1_) represents the probability that the user visits the i-th point after visiting the (i-1)-th point of the trajectory T, which is called the posterior probability. It is the probability that the attacker can obtain by analyzing, counting, and calculating the historical trajectory of the user, and it is an important manifestation of the user’s tendency. Thus, the propensity is quantified as the probability of the state transition in the mobile user trajectory, which is specifically expressed as P(T)∈[0,1], where 0 means no tendency and 1 means a strong tendency.

### 3.3 Problem characterization

If a user visits a location for the first time, there is a great probability that he will visit it again. Thus, it can be seen that the propensity of user’s activities can greatly reflect the behavior characteristics and life patterns. In this paper, a user’s propensity is measured by its probability of visiting a specific location point or the probability value of the corresponding Markov chain. In privacy protection, the propensity probability is used to measure the degree of privacy leakage, which must be expressed formally and expressed as a specific value, the propensity is measurable.

**Fig 2:**
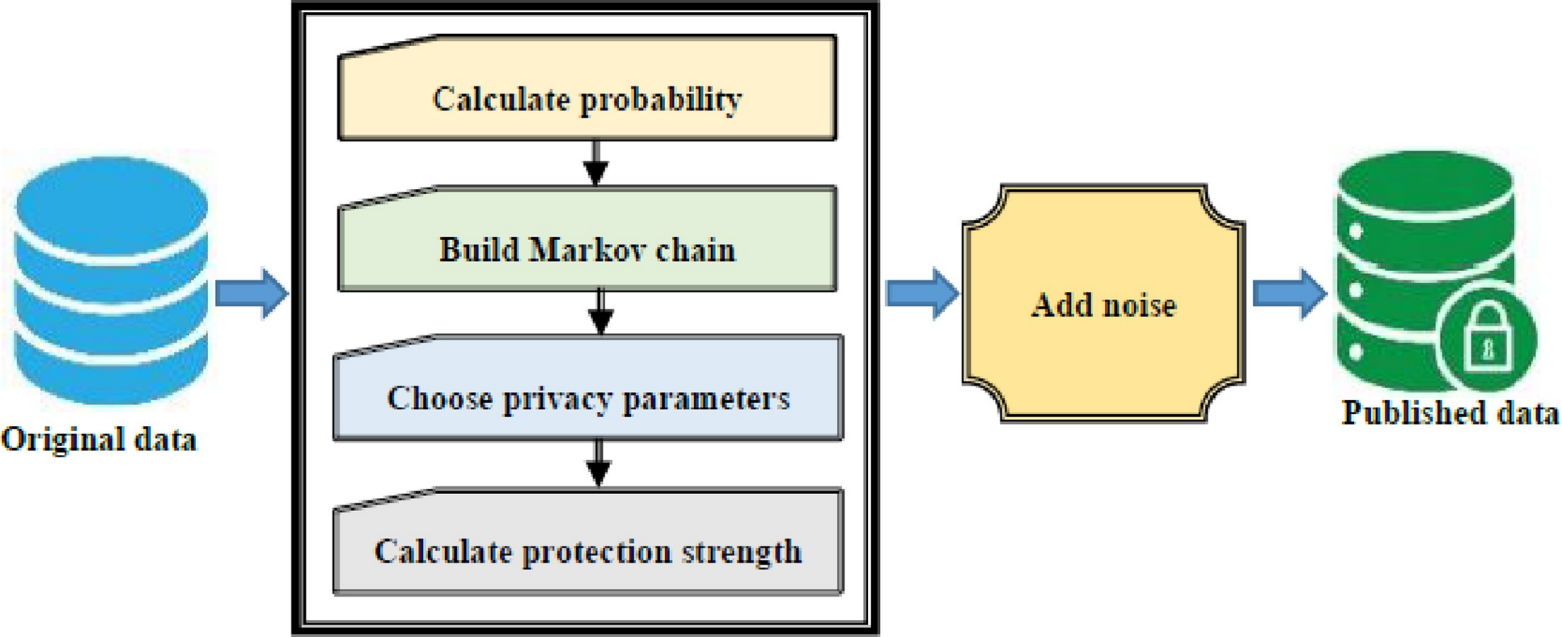
Workflow diagram.

It is not only necessary to protect the user’s location, trajectory and identity privacy, but also to protect the tendency of user, so as to effectively prevent the disclosure of his privacy information. However, the current research on user’s tendency protection has not been carried out in depth, how to use Markov model and differential privacy protection method to protect user’s tendency is the focus of this paper. The workflow of the proposed method as shown in Fig 2.

In this process, firstly, to calculate the probability of visiting each location point, and to calculate the state transition probability of position points and construct a Markov chain. Secondly, to select privacy protection parameters according to user needs, and to calculate the privacy budget parameters under different user tendency, and to add privacy protection noise. Finally, to publish anonymized trajectory data.

## 4. Proposed methodology

### 4.1 Markov model construction

From a statistical point of view, the Markov model is a widely used statistical model. The historical state closer to the present has a greater impact on the decision-making of the next state, while the earlier historical state can be ignored. If the user’s trajectory is related to the previous *k* states, a new trajectory will be generated, and the attacker cannot analyze the user’s original tendency from the new trajectory. Therefore, it is only necessary to use a first-order Markov model to deal with the user’s trajectory tendency.

According to the theory of probability theory and mathematical statistics, when the number of experiments is large, the probability can be measured by the frequency. Therefore, in this paper, the frequency of state transition is meant to approximately calculate the state transition probability. The method of calculating the transition frequency is used to calculate the state transition probability in the Markov model. The steps are as follows:

1. Perform preprocessing such as screening, filtering and segmentation based on historical trajectory data.
2. Calculate the initial frequency of state transition with the processed data to obtain the initial probability.
3. Calculate the frequency of state transition to obtain the state transition probability.
4. Construct a state transition matrix and build a Markov model.

As shown in Table 1, the state transition record is obtained by using the trajectory frequency calculation method, and *n*_*ij*_ is the transition times from the current state *s*_*i*_ to the future state *s*_*j*_. By using the number of state transitions, the frequency of state transitions can be reached.

**Table 1:** Frequency table of state transitions.

Current state	Future state					
Frequency	s_1_	s_2_	…	s_j_	…	s_n_
s_1_	n_11_	n_12_		n_1j_		n_1n_
s_2_	n_12_	n_22_		n_2j_		n_2n_
…	…	…	…	…	…	…
s_j_	n_j1_	n_j2_		n_jj_	…	…
…	…	…	…	…	…	…
s_n_	s_n1_	s_n2_	…	s_nj_	…	s_nn_

For subsequences {x_0_,x_1_,x_2_,…,x_i_,x_j_,…,x_n_}, *f*_*kij*_ is used to represent the frequency of transition from state *i* to state *j*, *i*,*j*∈E, then the transition frequency matrix of time interval Δt for:

$$f_{kij}=\left[\begin{array}{cccc} f_{k11} & f_{k12} & \cdots & f_{k1n}\\ f_{k21} & f_{k22} & \cdots & f_{k2n}\\ \vdots & \vdots & \ddots & \vdots \\ f_{kn1} & f_{kn2} & \cdots & f_{knn} \end{array}\right]$$ (12)

Substituting frequency for probability, the transition probability for time interval Δt is estimated as:

$$F_{kij}=\frac{f_{kij}}{\sum_{j=1}^{n}f_{kij}}$$ (13)

Then the state transition probability with interval Δt is:

$$P_{ij}=\left[\begin{array}{cccc} p_{11} & p_{12} & \cdots & p_{1n}\\ p_{21} & p_{22} & \cdots & p_{2n}\\ \vdots & \vdots & \ddots & \vdots \\ p_{n1} & p_{n2} & \cdots & p_{nn} \end{array}\right]$$ (14)

The core of establishing a user-propensity Markov model is to calculate the prior probability of a location point and its transition probability. Let T be the set of user trajectory data, any trajectory can be represented as *t*_*i*_ (*t*_*i*_∈T), then the prior probability of any point *l*_*i*_ (*l*_*i*_∈t) on the trajectory *t*_*i*_ is:

$$\text{P}\left(l|t_{i}\right)=\frac{num\left(l_{i},t_{i}\right)}{num\left(t_{i}\right)}$$ (15)

Where num(*l*_i_,t_i_) represents the occurrence of location point *l*_*i*_ in trajectory *t*_*i*_, and num(*t*_*i*_) represents the occurrence of location points in the trajectory *t*_*i*_.

All user’s trajectories are divided into sub-trajectory, which contains only two adjacent nodes. The sub-trajectories are connected to synthesize synthetic trajectories after processing. Each node in the sub-trajectory is mapped to each state in the Markov model, and the transition of any two adjacent states are corresponded to the transition of position point in the trajectory sequence. The propensity probability is a conditional probability that can be expressed as the quotient of the number of specific trajectories and the total number of trajectories. The transition probability of two adjacent nodes *l*_*i*_ to *l*_*j*_ can be expressed as the propensity probability. The state transition matrix is composed of the state transition probabilities of all trajectories. The specific method is shown in Algorithm 1.

**Table:** 

Algorithm 1: Calculate the probability of state transition
Input: Trajectory data set T, *t*_*i*_ is any trajectory in T, *l*_*i*_ is any position point on *t*_i_.
Output: State occurrence probability P(*l_i_*/*t_i_*) and state transition probability P(T).
1 The trajectory sequence T is divided into sub-trajectory sequences *t*_*i*_ which contains only adjacent nodes
2 Every interval Δt time (each round):
3 for each t_i_∈T do
4 Calculate the occurrences num(*t*_*i*_) of the trajectory *t*_*i*_ in T
5 for each *l*_*i*_∈*t*_*i*_ do:
6 Calculate the occurrences num(l_i_,t_i_) of the trajectory *l*_*i*_ in *t*_*i*_
7 end for
8 end for
9 Calculate the prior probability of any point *l*_*i*_ on any trajectory t_i_: $\text{P}\left(l_{i}|t_{i}\right)=\frac{num\left(l_{i},t_{i}\right)}{num\left(t_{i}\right)}$
10 Calculate the state transition probability in the mobile user trajectory: $P(T)=P(l_{1})\prod_{i=2}^{n}\text{p}\left(l_{i}|l_{i-1}\right)$
11 Return the state occurrence probability P(*l*_i_|t_i_) and the state transition probability P(T).

The main idea behind the algorithm is to calculate the access probability and the state transition probability by calculating the state transition frequency that finds the state transition probability in the Markov model. The trajectory sequence T is divided into a set of sub-trajectory sequences *t*_*i*_ which only contains adjacent nodes, and each node of the sub-trajectories is mapped to each state in the Markov model. First, the frequency of occurrence of subsequences is calculated, then the frequency of occurrence of each node is calculated, then the access probability and the transition probability are calculated, and finally the Markov model is constructed. Each loop calculates the number of trajectories and the position points on the trajectory is executed *n* times, so the time complexity of the algorithm is O(n^2^). In the Markov model, the transition probability of various states is a reflection of a user’s tendency to visit certain locations. Therefore, a user’s propensity has a great correlation with the probability of state transitions in Markov chains, and the trajectories with high propensity and high risk of privacy leakage should be protected.

### 4.2 Extended differential privacy protection model

A new (P_i_,ε_i_)-differential privacy protection model is proposed to protect user’s tendency privacy based on the constructed Markov chain. It combines the user’s state transition probability p_i_ and differential privacy budget parameter ε_i_, and it dynamically adjusts ε_i_ according to p_i_, so as to differentially add Laplacian noise, to dynamically protect the user’s tendency privacy, and to improve the data availability. The specific description is as follows:

***Definition 5*:** (P_i_, ε_i_)-differential privacy protection model. If *p*_*i*_ is the propensity probability of user *u* transitions from a state to the state *i*, its corresponding differential privacy budget parameter *ε*_*i*_ is satisfied with the following conditions, then the differential privacy protection model is called (P_i_, ε_i_)-differential privacy protection model

$$\varepsilon_{i}=\frac{R}{p_{i}}$$ (16)

Where *R* is the privacy model parameter, which is a constant. R∈(0,1], it is divided into 10 privacy levels: A, B, C, D, E, F, G, H, I, J, where the corresponding values are: 0.1, 0.2, 0.3, 0.4, 0.5, 0.6, 0.7, 0.8, 0.9, and 1.0. Level A is the most sensitive and level J is the least sensitive. The R value is selected by a user or the system based on privacy protection requirements. The term *p*_*i*_ is the tendency probability of a user visiting a state *l*_*i*_, that corresponds to the state transition probability of the Markov chain, *p*_*i*_∈(0,1]. *ε*_*i*_ is the privacy budget parameter corresponding to the user in state *l*_*i*_, *ε*_*i*_∈(0,+∞).

It can be seen from Eq 16, for a constant R, the larger state transition probability *p*_*i*_ is, the smaller privacy budget parameter *ε*_*i*_. Thus, the more noise is added, the better the privacy protection effect is. When *p*_*i*_ = 1, the privacy differential privacy budget parameter is a fixed constant, i.e., it does not change with the user’s tendency. Thus, the (P_i_,ε_i_)-differential privacy protection model is a traditional ε-differential privacy protection model. By introducing the privacy model parameter R, the differential privacy budget parameters can be changed according to a user’s tendency, and differential disturbance noise can be added to achieve dynamic adjustment and change the differential privacy protection. The model can effectively solve the problem of user tendency leakage in trajectory data, the specific execution process is shown in Algorithm 2.

**Table:** 

Algorithm2: (P_i_,ε_i_)- Differential privacy protection algorithm
Input: Markov chain P(T), Privacy threshold θ, Privacy model parameter R
Output: Noise-added trajectory sequence L(D)
1 for i = 1 to n do
2 P_i_ = P(T_i_)
3 if P_i_ >θ {
4 ε_i_ = R/P_i_
5 ε = ε∪ {ε_i_ }
6 $L(D_{i})=f(D_{i})+Laplace(\frac{△f}{\varepsilon_{i}})$
7 LD = LD∪ {LD_i_ }
8 end if }
9 end for
10 return L(D)

The premise of the algorithm is to combine user’s tendency and a privacy model parameter, to calculate and set the differential privacy budget parameters and add a differential Laplacian noise, which can effectively prevent tendency attacking. The larger the user’s state transition probability P_i_ is, the smaller the allocated privacy budget ε_i_ is. The more noise is added, the higher degree of privacy protection is. Therefore, in this algorithm, after the undirected graph is constructed by the state transition matrix, when each vertex is connected to other vertices, the algorithm executes the most times in the undirected graph, so its time complexity is O(n(n-1)).

A dynamic balance is achieved between privacy protection and data utilization by correlating the tendency *p* and the privacy budget parameter *ε* with the privacy protection parameter *R*. If a better privacy protection effect is required, a smaller *R* value is selected, then more noise is added which improves the privacy protection effect and reduces the data utilization rate. However, if a better data utilization rate is required, a larger R value is selected, which adds less noise and reduces the privacy protection strength to improve the data utilization rate. The specific process is divided into five steps:

***Step 1***: Calculate the probability of location point. According to the user’s movement trajectory, it calculates the probability of visiting the location points, and it analyzes statistics and calculates the probability of the user transitioning of these state points.
***Step 2***: Build the Markov chain. It uses the weight of the state point to represent its strength in the cluster set, it calculates the number of transitions of states and normalizes it to the transition probability of different states in the Markov chain, and finally it to construct the Markov chain.
***Step 3***: Calculate privacy budget parameters. According to the privacy protection parameter R and the tendency probability P_i_, it combines with the (P_i_,ε_i_)-differential privacy model conditions, the privacy budget ε_i_ is calculated which is correspond to the position point on the trajectory.
***Step 4***: Anonymize the trajectory data. According to the ε_i_ of different states, the Laplacian noise is dynamically added to generate the released trajectory sequence.
***Step 5***: Trajectory data release. Publish the final trajectory sequence to use by third parties.

### 4.3 Measurement of privacy protection effectiveness

#### 4.3.1 The measurement of information leakage

Information leakage (IL) is mainly measured by the degree of information leakage of original data in the published data. The smaller the degree of information leakage is, the stronger the privacy protection capabilities is. The degree of information leakage can be expressed as the ratio of correct matching of data records in the published data set and the original data set [37], which is specifically calculated as:

$$IL=\frac{\sum_{t\in D}\mathrm{Pr}\left(t^{\prime }\right)}{n}$$ (17)

Thus, *n* represents the number of data records in the original data set, and *Pr(t’)* represents the published data record [38], which can be calculated as:

$$\mathrm{Pr}\left(t^{\prime }\right)=\left\{\begin{array}{c} 0\;\;\;\;\;\;\;\;\;\;\;\;\;,t\notin G\\ \frac{1}{\left|G\right|}\;\;\;\;\;\;\;\;,t\in G \end{array}\right.$$ (18)

*G* is a cluster set which is contained in the original data record.

#### 4.3.2 Measurement of data availability

Data availability (DA) mainly refers to the efficiency of published trajectory data for a third party. It also reflects the loss rate of trajectory information during the anonymization process, which is generally measured by calculating the amount of information loss in published data. In this paper, the standard deviation of distance error between the position on the real trajectory and the corresponding position on the published trajectory is used to measure the distance error. The more amount of information loss in the trajectory, the lower availability of trajectory data. The specific calculation is:

$$\text{DU}=\sqrt{\frac{\sum_{i=1}^{n}{\left(d_{i}-\bar{d}\right)}^{2}}{\left|L\right|}}$$ (19)

Where *d*_*i*_ represents the Euclidean distance between the position *loc*_*i*_ on real trajectory and the corresponding position point on published trajectory, $\bar{d}$ represents the average distance between the real trajectory and the corresponding position point on the published trajectory, *n* represents the number of position points on the trajectory, and *|L|* represents the length of trajectory.

## 5. Experimental evaluation and results analysis

The proposed method was evaluated experimentally in terms of privacy protection effect, data availability and execution efficiency, and the feasibility and effectiveness of the proposed method are evaluated through experiments. The proposed method (referred to from here on as the (P, ε)-DP) was compared against the standard differential privacy protection method (referred to from here on as the DP) and the method proposed in paper [39] (referred to from here on as the α-DP).

### 5.1 Experimental design and parameter setting

The experiments are conducted on a computer with an Intel Core (TM) i5-3470 CPU @ 3.2 GHz and 8 GB RAM running over the Microsoft Windows 7.sp1.64 bit operating system. The algorithm is implemented in Python. The experimental data comes from the Geolife data set [40–42], which collects GPS trajectory data of 182 users over 5 years. The data set is represented by a time-stamped point sequence, each point contains latitude, longitude, Elevation, date and time information, it contains 17,621 trajectories, a total distance of 1,292,951 kilometers, and a total duration of 11,129 days. Among them, 91.5% of the trajectories are in line with the dense representation, and the data includes various outdoor activities in the user’s daily life, such as going to work and going home in daily life, as well as some recreational activities and sports activities, such as shopping, sightseeing, dining, hiking and cycling bicycles and more. In the paper, 1,000 trajectories of a certain area are sampled from this dataset, and approximately 1,346,480 location points are formed after sampling. In the experiments, the original data is first processed in batches to separate the user’s longitude, latitude, altitude, date and time, and it uses the GIS online conversion tool (https://www.mygeodata.cloud/) to resolve the user’s latitude and longitude into the actual address, Table 2 shows the processed data set.

**Table 2:** Experimental data set.

Longitude	Latitude	Altitude	Date	Time	Address after resolution
116.3266533	39.9924916	0	2008/3/20	10:03:40	37 North Fourth Ring West Road, Haidian District, Beijing
116.3305399	39.9753799	0	2008/3/24	17:40:10	18 Shuangyushu Xili, Haidian District, Beijing
116.3277733	39.9752816	0	2008/4/2	12:01:06	Courtyard 46 Zhongguancun Street, Haidian District, Beijing
116.4228966	39.9704599	0	2008/4/2	18:49:24	Building 5, Yard 3, Xiaohuangzhuang, Chaoyang District, Beijing
116.328001	39.976452	183	2008/4/22	1:58:55	40 Zhongguancun Street, Haidian District, Beijing
116.329016	39.97542	188	2008/4/22	2:09:35	Unit 9–12, Yard 46, Zhongguancun Street, Haidian District, Beijing
116.378501	39.966715	124	2008/4/22	11:37:43	Gate 1–3, Yard 10, Xinjiekou Outer Street, Xicheng District, Beijing
116.41839	39.969297	178	2008/4/22	13:03:31	Building 3, Yard 6, Xinghua Road, Dongcheng District, Beijing
116.35138	39.966527	149	2008/4/22	14:35:40	Building 6, Courtyard 39, Xueyuan South Road, Haidian District, Beijing
116.327536	39.975453	145	2008/4/27	13:11:21	42 Zhongguancun Street, Haidian District, Beijing

Where the 1st and 2nd columns represent the latitude and longitude coordinates of the user’s current location, the 3rd column represents the altitude of the user’s location, the 4th and 5th columns represents the time and date of the user’s trip, and the 6th column represents the actual address that has been translated. Then it uses the Markov clustering algorithm to extract the user’s state points, build the user’s Markov chain, analyze and calculate the user’s tendency. Finally, the (P_i_,ε_i_) differential privacy protection model is used to reduce the sensitivity of differential privacy, dynamically add Laplacian noise to control user’s tendency and protect his privacy.

### 5.2 Experimental results and analysis

#### 5.2.1 Analysis of privacy protection effect

Privacy protection effect mainly refers to the effect that user’s tendency in trajectory data is protected or hidden, it also refers to the disclosure degree of user’s tendency privacy information. The smaller disclosure degree of sensitive information is, the stronger privacy protection ability is. In practice, the intensity of privacy protection is indirectly reflected by differential privacy budget parameters. ε value is smaller, the more noise is added, and the implementation of the privacy protection level will be higher.

**Fig 3:**
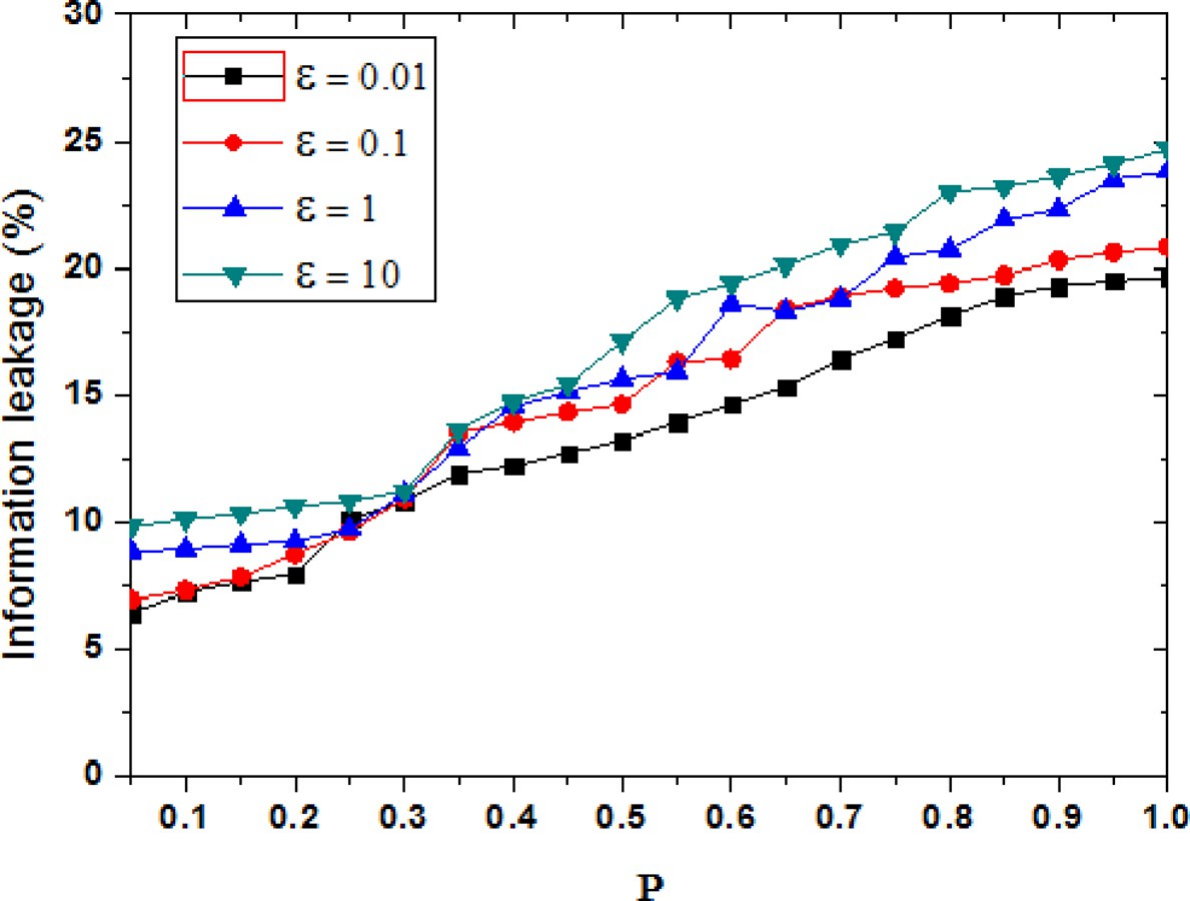
Comparison of information leakage under different parameters.

Fig 3 shows the privacy protection effect comparison of the proposed methods when the privacy budget parameter ε is set to {0.01, 0.1, 1, 10}. When the user’s tendency probability P is given, the smaller the privacy budget parameter is, the higher the degree of privacy protection is, and the degree of information leakage is small. With the value of privacy protection parameter ε is added, the difference privacy method decreases the disturbance degree of the original trajectory data, and the privacy protection level also is decreased. The degree of information leakage continues to increase, whereas the privacy protection effect continues to decrease. When the value of ε is small, more protection noise is added. The smaller the degree of information leakage, the better privacy protection effect is. As can be seen from Fig 3, when ε = 10, the average information leakage degree is significantly higher than the other three cases. With the increasing of probability P, the probability of user visiting a specific location is raised and the user’s tendency is raised, the sensitive information contained is added, if the amount of added noise is constant, the information leakage will be increased.

R is the privacy model parameter, which is selected by the user or system according to the privacy protection requirements. When R is increased, it means that the privacy protection requirement of the user is reduced, and the added noise is reduced in the data set, so the degree of disruption of the real data set will be reduced, and the degree of privacy protection will be reduced. As a result, the level of privacy information leakage is increased. According to the formula 16, when privacy budget parameter ε is a constant, the parameter P is increased, the parameter R will be increased. However, the added noise of data set is not increased, the actual privacy information leakage risk will be increased further.

**Fig 4:**
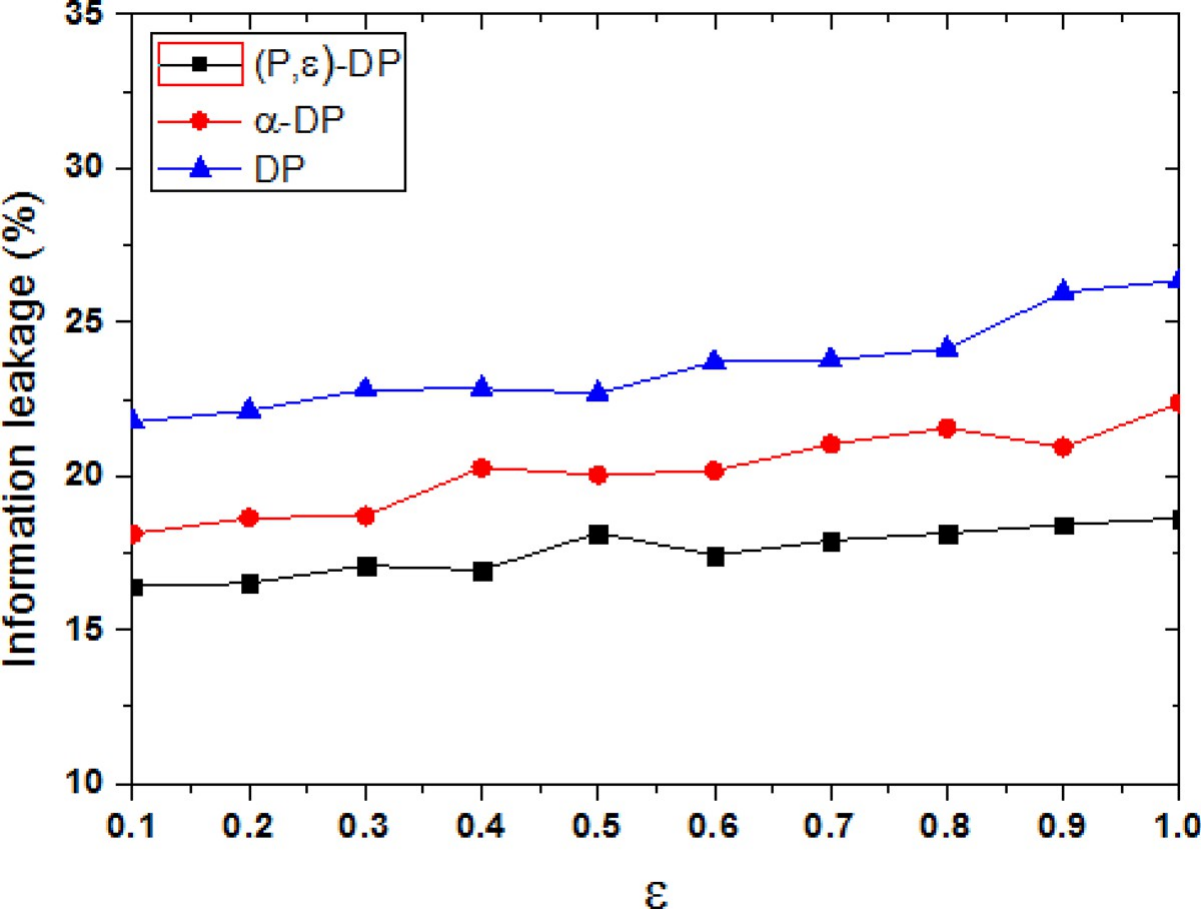
Comparison of information leakage under different methods.

With the increase of privacy protection budget value, the disturbance of the original trajectory data is reduced by the differential privacy protection method, and the privacy protection level is reduced. Fig 4 shows the comparison of information leakage degree between the proposed method and the other two methods, the privacy model parameter is P = 0.6. Privacy protection is inversely proportional to the privacy budget ε, smaller ε means larger data distortion is, and the higher the degree of privacy protection is. When ε tends to zero, the privacy protection reaches the highest level in theory. As can be seen from Fig 4, it shows that the degree of information leakage of the three methods increases as the value of ε increases. When the value of ε is added, the protection noise is relatively reduced, which means that the possibility of sensitive information leakage will be increased.

The privacy information leakage degree of DP method is the highest, because it uses the privacy budget to randomly add noise, the added noise is distributed independently and uniformly, it is low correlation with the original track data. The attacker can reduce the interference caused by noise through filtering, so the effect of privacy protection is relatively low. The privacy information leakage degree of α-DP method is also higher, although the added noise data by α-DP method is time-dependent with user trajectory data, there is no specific protection for user’s tendency privacy. However, the privacy information leakage degree of the proposed (P,ε)-DP method is the lowest, it quantifies the probability of user’s tendency, and it dynamically adds different noise. It is more targeted to protect user’s tendency, so it achieves the lowest degree of information leakage. When the propensity probability is a constant, it can adjust the privacy-preserving strength according to the privacy model parameters. Compared with other methods, the information leakage of the proposed method is reduced by 13.03%、25.65% on average.

**Fig 5:**
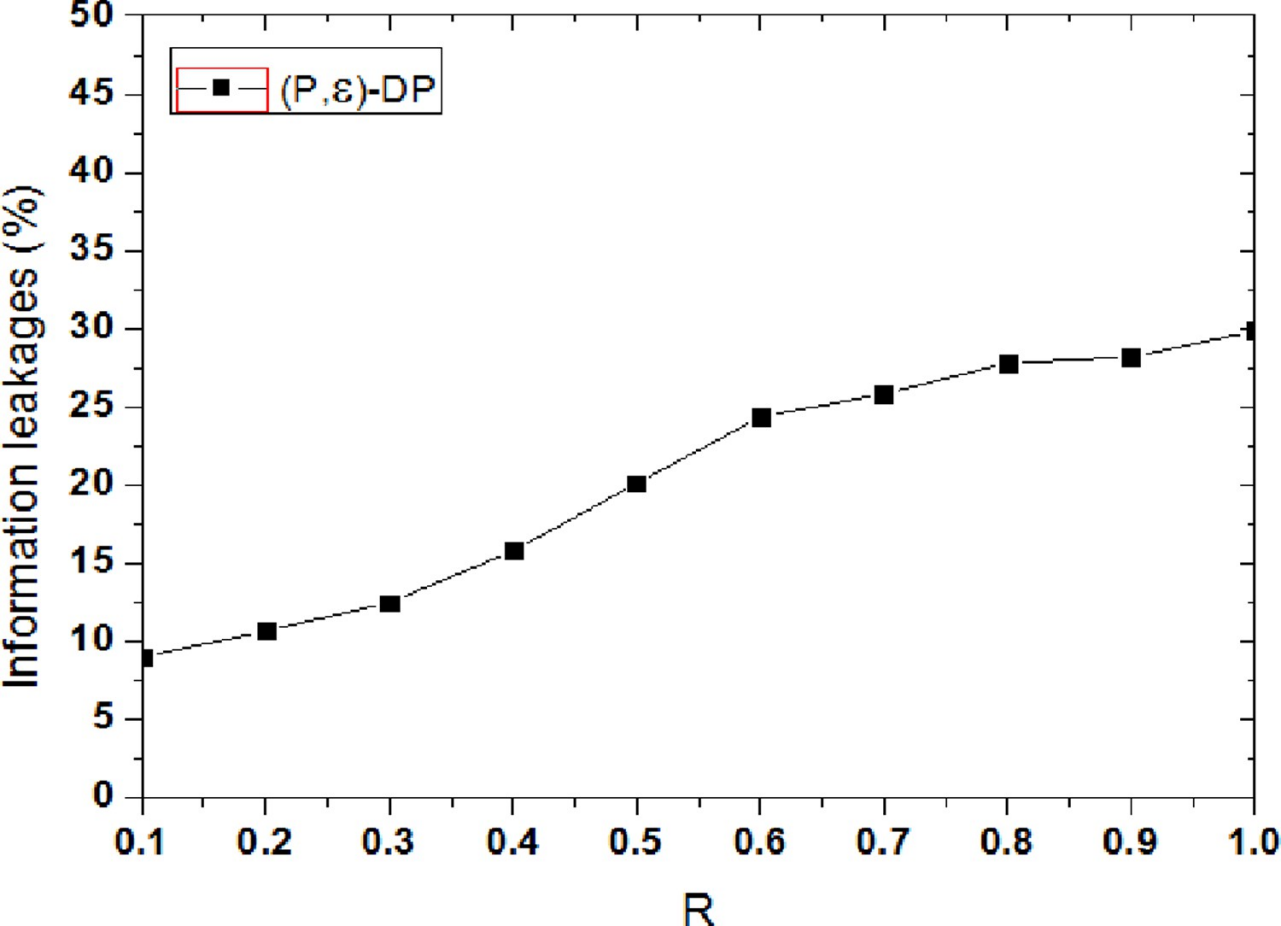
Effect of privacy model parameter on information leakage.

Fig 5 shows the influence of privacy model parameters on information leakage degree when ε = 0.01. When the privacy model parameter R is increased, the degree of leakage of user sensitive information is also expanded. Because R is added, it means that the user’s privacy protection requirement is reduced, then the added noise is reduced in the data set, and the degree of privacy protection of real data is reduced, therefore, the disclosure of private information is increased. Meanwhile, according to the Eq 16, the privacy budget parameter ε is a fixed constant, when the value of R is increased, the value of P is also increased, the tendency of user to visit a certain location becomes more obvious. However, the added noise is not increased in the data set, so the actual risk of privacy information leakage is further increased.

#### 5.2.2 Analysis of data availability

**Fig 6:**
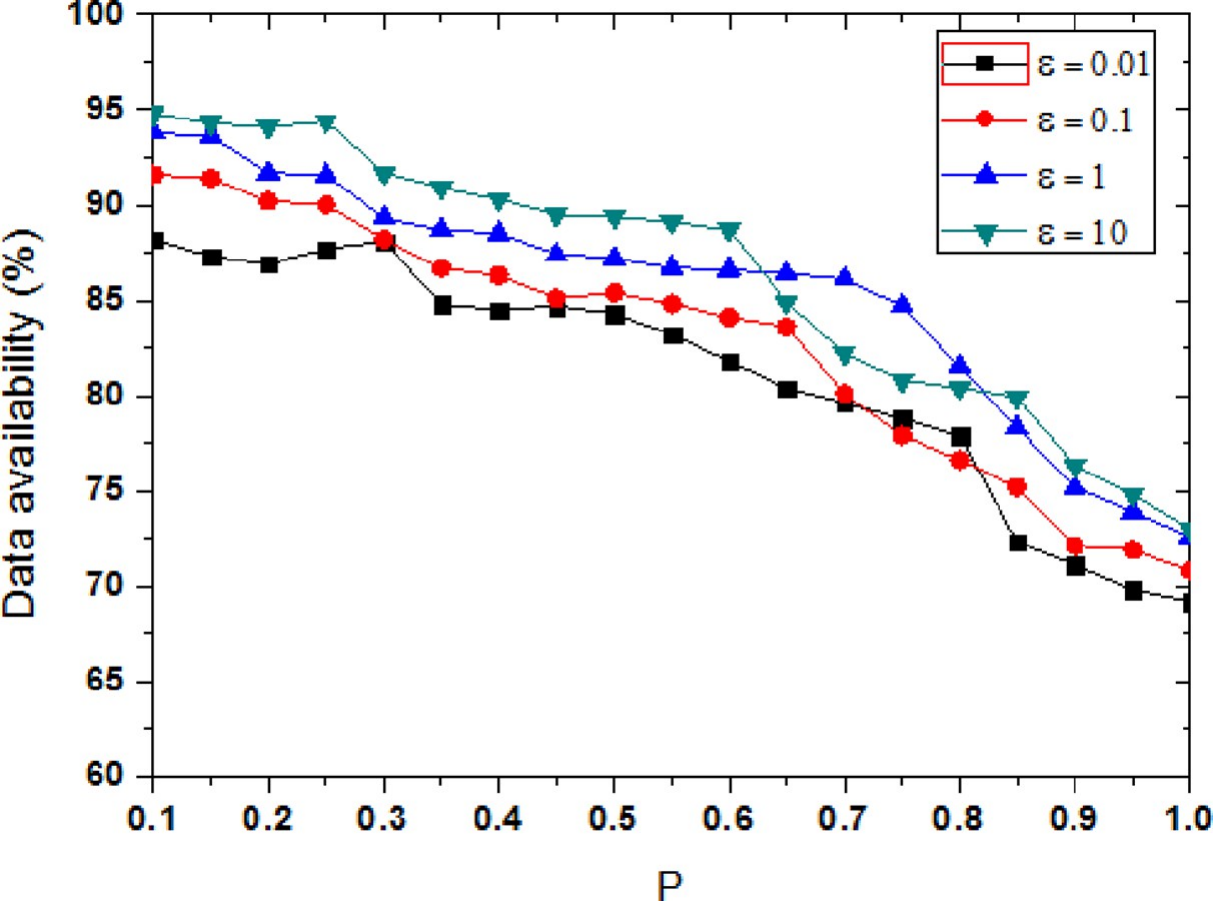
Comparison of data availability under different parameters.

Data availability refers to the efficiency which published trajectory data is used by third parties, and it also indirectly reflects the rate of loss of trajectory information during anonymization. Fig 6 shows the data availability comparison of the proposed methods when the privacy budget parameter ε is set to {0.01, 0.1, 1, 10}. For the different privacy protection parameters ε, the data availability is not the same. When the value of ε is small, the added protection noise is relatively large, and the data availability is relatively low. When ε = 0.01, its average data availability is significantly lower than the other three cases. As the user visiting probability P is increased, the probability of the user visiting a specific location is increased, and the sensitivity of data is raised. To protect user’s tendency privacy, the amount of added noise will continue to increase, which leads to data availability is decreased.

According to the constraints in the (P,ε)-differential privacy protection model, when the privacy model parameter R is a constant, the user’s propensity probability P is inversely proportional to the privacy budget, and the larger value of P is, the smaller value of ε is. In this case, the more disturbing noise is added, the availability of data is reduced. The proposed (P,ε)-DP method can flexibly assign certain privacy preserving model parameter according to the user’s propensity probability. When the privacy parameter is fixed, the larger the propensity probability is, the smaller the corresponding privacy budget is, and the added noise will be increased, so the availability of data is reduced

**Fig 7:**
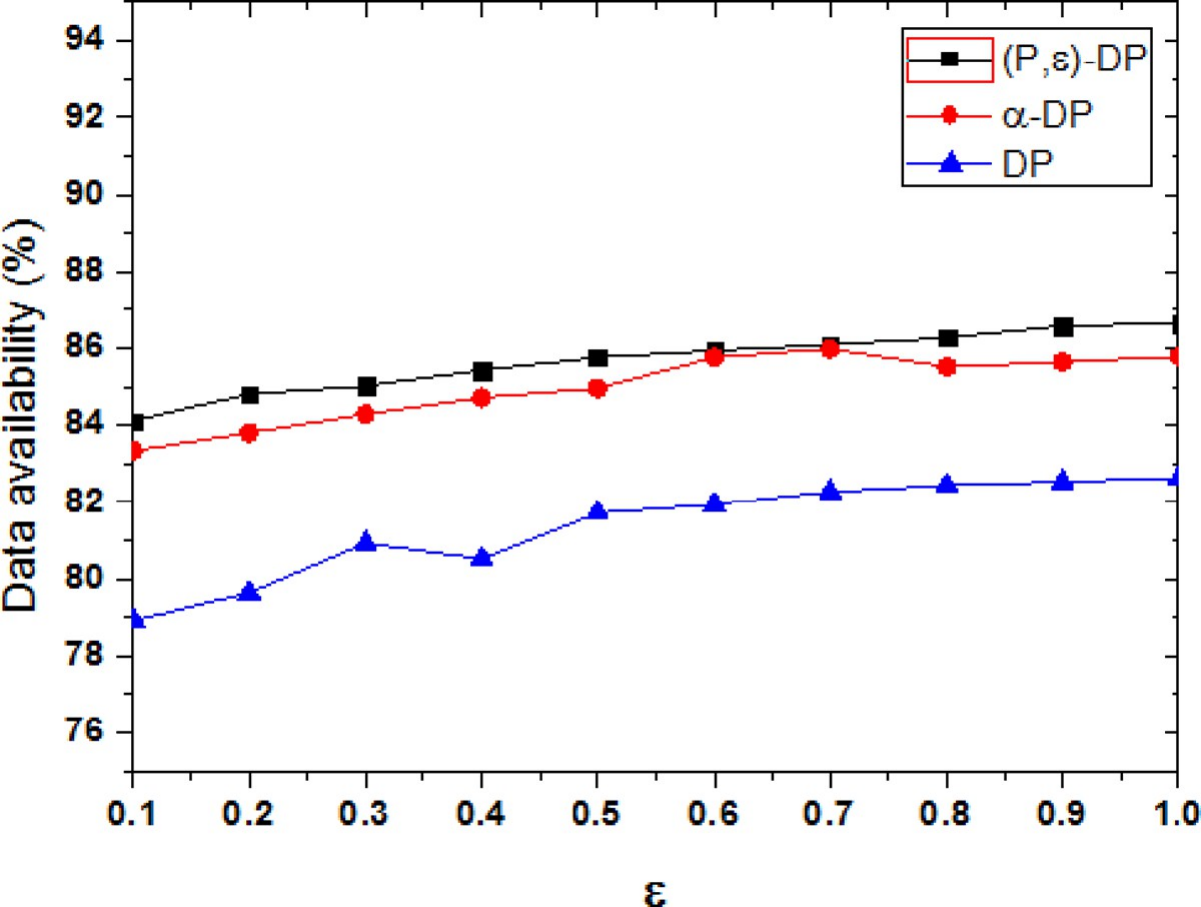
Comparison of data availability under different methods.

Fig 7 shows the comparison of the data availability between the proposed method with the other two methods when the privacy model parameter is P = 0.6. ε is a key parameter in differential privacy, it is used to determine the strength of privacy protection and the amount of added noise. In the experiment, the availability of data is tested with different ε, when the value range of ε is [0.1, 1] and the step size is 0.1. From the comparison of different ε values, the data availability of three methods are increased as the value of ε is added. When the value of ε is increased, the amount of added noise is relatively reduced, and data availability is increased.

From the comparison of different methods, the DP method randomly adds the same noise so that the data availability is the lowest. Both α-DP and (P, ε)-DP method can add different noise, and the added noise is targeted, so that the availability of the data is higher. However, α-DP method cannot quantify the user’s tendency, it adds relatively more noise, so the usability of the data is lower than the (P, ε)-DP method. The (P, ε)-DP method can dynamically adjust the added disturbing noise through the privacy preserving parameter R and the propensity probability P, and the added noise is more accurate, so its data availability is the highest. Compared with other methods, the data availability of the proposed method is improved by 0.81% and 5.29% on average.

**Fig 8:**
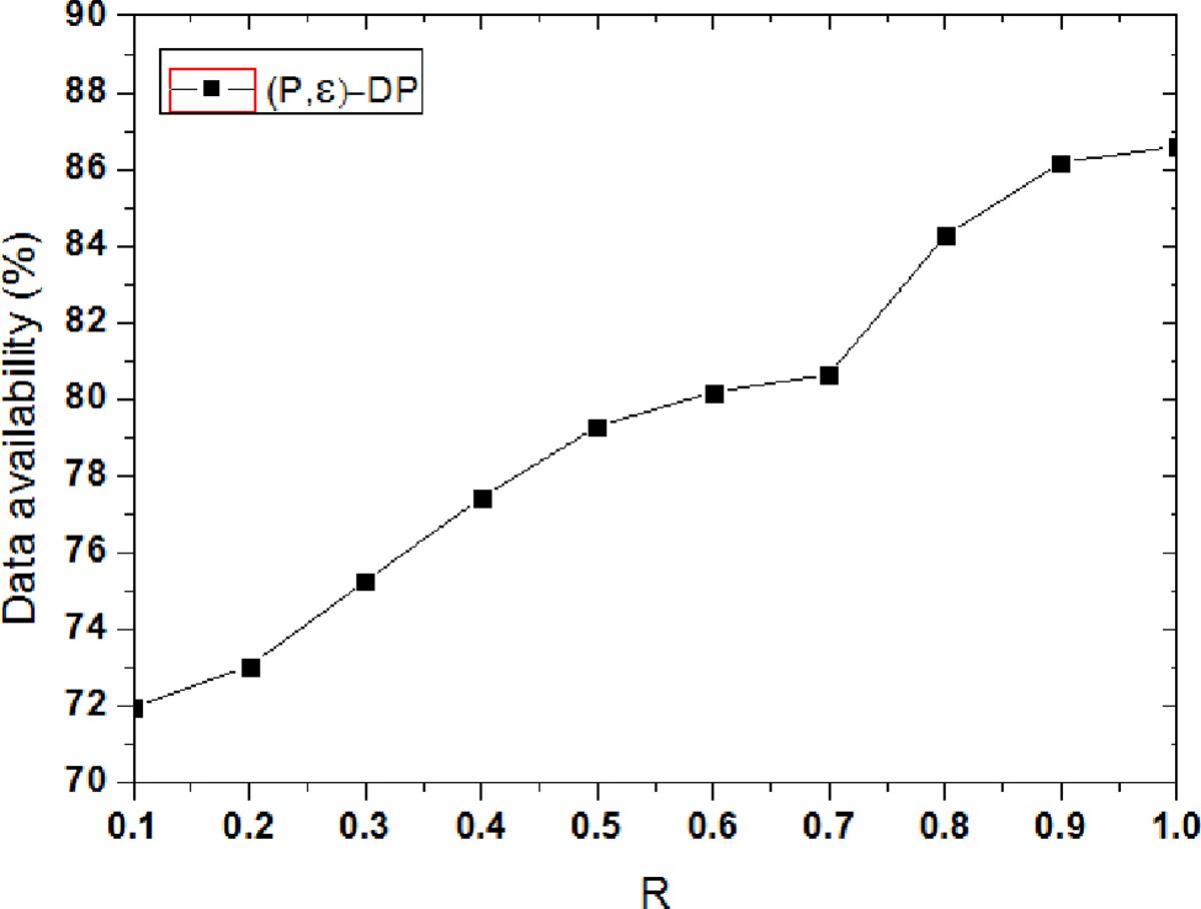
Effect of privacy model parameter on data availability.

Fig 8 shows the influence of privacy model parameters on data availability. Thus, when ε = 0.01, with the privacy model parameter R is increased and the availability of data shows an upward trend. Because R is added, it means that the user’s privacy protection requirement is reduced, then the added noise is reduced in the data set, and the information loss of the data set is reduced, therefore, the availability of data is increased. According to the constraints in the (P, ε)-differential privacy protection model, when the privacy budget parameter ε is a fixed constant, the value of R is increased, then the value of P is also increased. At this time, the tendency of users to visit a certain location becomes more obvious. However, the added noise is not increased in the data set, so the data availability is improved. It can be seen from the experiment, when R = 0.1, the data availability is 72.13%, when R = 1, the data availability is 86.39%.

#### 5.2.3 Analysis of execution efficiency

**Fig 9:**
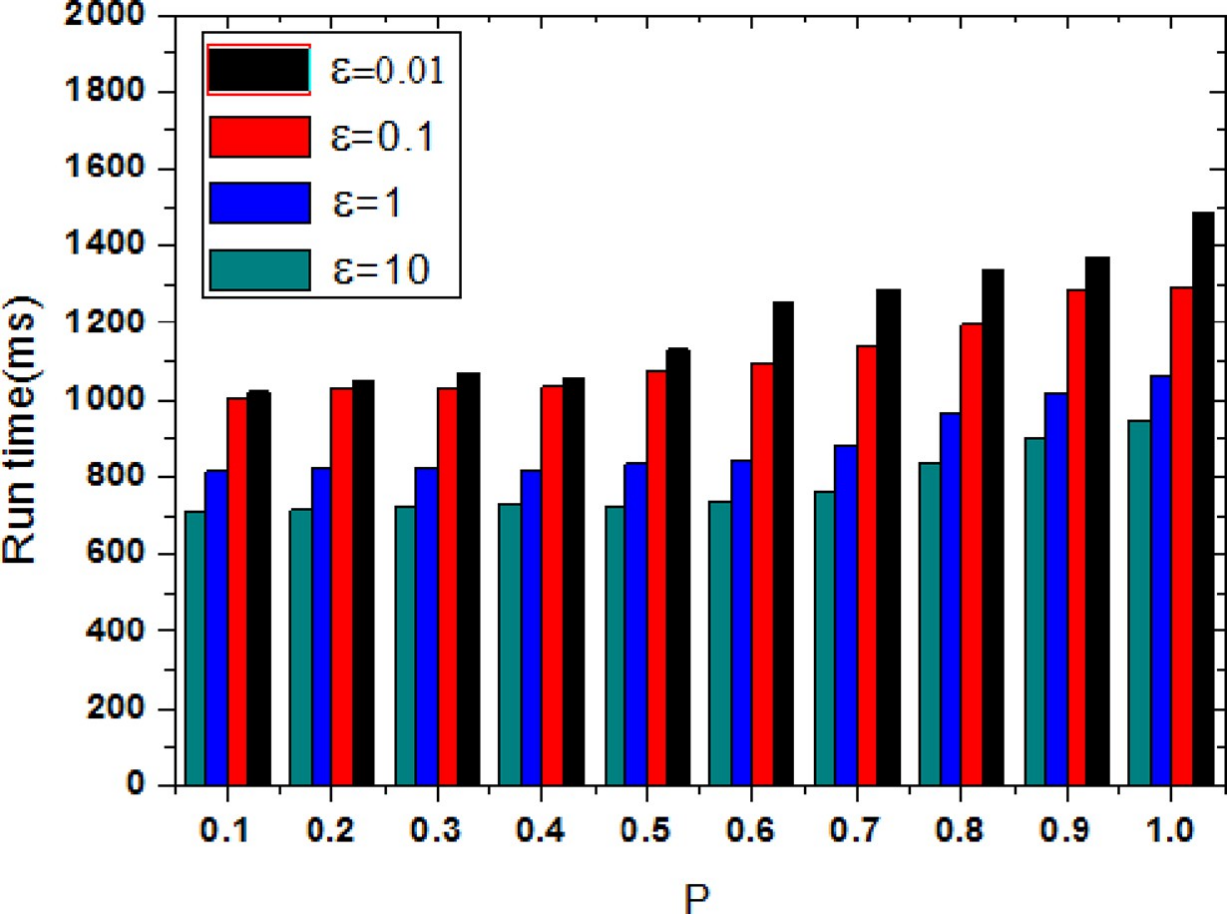
Comparison of execution efficiency under different parameters.

Execution efficiency is the time complexity of execution; it is mainly measured via the execution time. In Fig 9, the execution efficiency comparison of the proposed methods when the privacy budget parameter ε is set to {0.01, 0.1, 1, 10} is shown. It shows that the execution time for each of the four parameters is not the same; instead, the execution time is proportional to the added noise. The more noise is added, the longer the execution time is. When the probability of a user visiting a specific location is less than 0.5, the user’s sensitive information is relatively small, the difference of added noise via four privacy protection parameters is not large and the impact of different privacy parameters on execution time is not obvious. When the user visiting probability is increased, sensitive information is also gradually increased; thus, as the amount of added noise keeps increasing, and the execution time also becomes larger. Additionally, when ε = 0.01, the average execution time is significantly higher than the other three cases. When the user visiting probability P value is added, a user’s probability of visiting a specific location is raised, and his tendency is increased. To protect his tendency privacy, the amount of added noise will gradually grow, and the execution time will continue to enlarge.

**Fig 10:**
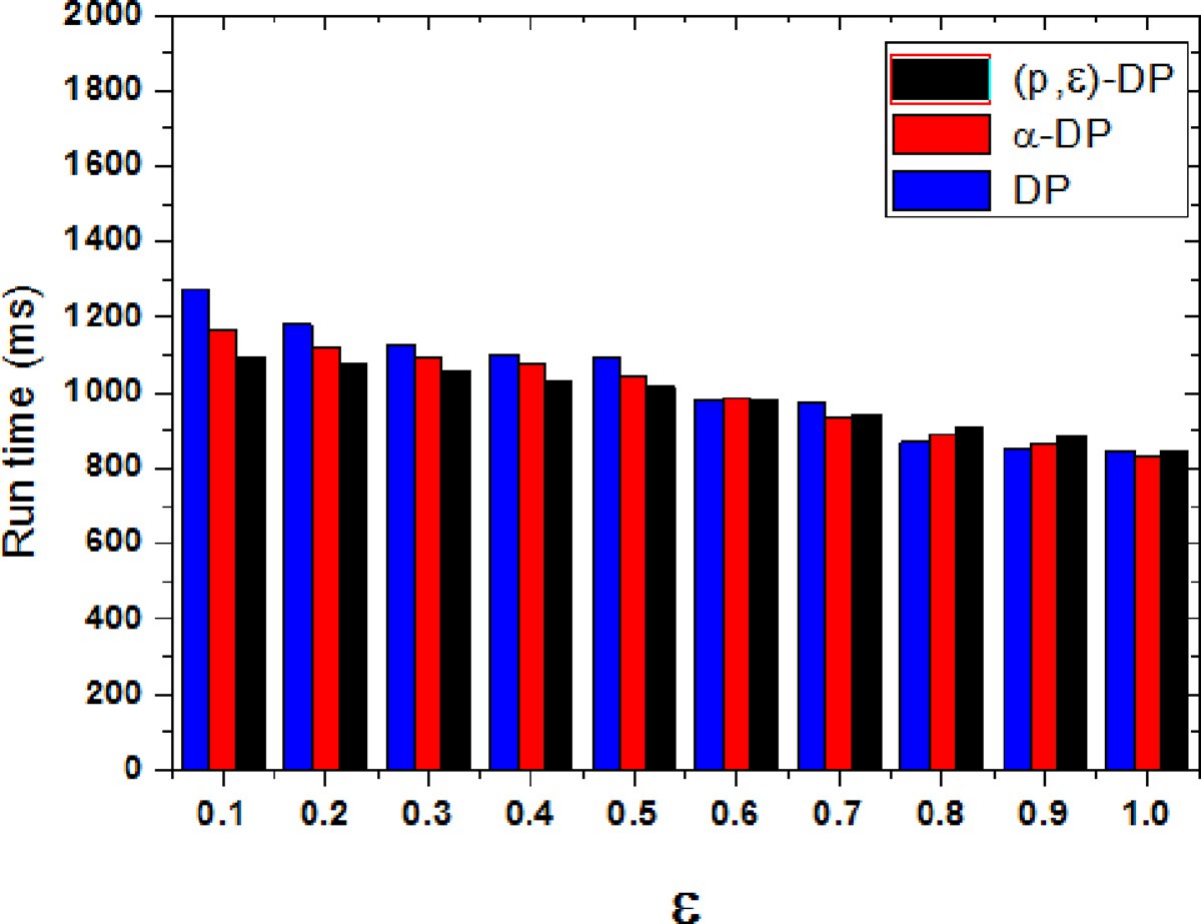
Comparison of execution efficiency under different methods.

Fig 10 shows the comparison of execution efficiency between the proposed method and two other methods when the privacy model parameter is P = 0.6. The execution time is closely related to the time complexity and calculation complexity of the algorithm. The comparison of various ε values show that the execution time of three methods is decreased with increasing ε. When the value of ε is increased, the added noise and the execution time are both relatively reduced.

When ε is small, the added disturbance noise is relatively more, and their privacy preservation effect is better. The efficiency of proposed method is slightly higher than the other two methods. When the user’s tendency is large, it needs to add more noise, while the proposed method can accurately add an appropriate amount of noise according to the quantized tendency probability. The other two methods cannot quantify the user’s tendency, and they need to add more noise to achieve the same privacy protection effect, so their time complexity are relatively high. As the ε is increased, the less noise is added, there is little difference in the execution efficiency of the three methods. Because the added disturbing noise is almost the same when the user’s propensity protection requirement is not strong, their time complexity is also basically the same. As can be seen from the experiments, the execution time of the proposed method is reduced by 1.71% and 4.48% on average.

#### 5.2.4 Experimental results

The experiments discussed above altogether indicated that the proposed method can provide efficient privacy protection for user’s tendency privacy and ensure high availability for published trajectory data., and its execution efficiency has been improved. The user’s tendency probability is quantified by Markov model, by constructing Markov chain, the user’s tendency probability is converted into the state points on the Markov chain, so that it can use the Markov model to deal with the user’s tendency to visit a specific location, it can improve privacy effectively. The user’s tendency is dynamically protected by differential privacy model, the user’s tendency and differential privacy budget parameters are correlated, which makes it possible to dynamically add noise according to the user’s tendency probability to provide personalized privacy protection, it can significantly improve data availability.

## 6. Conclusion

If a user visits a location for the first time, there is a great probability that he will visit it again. To solve the problem of privacy protection based on user’s activity rules and tendencies in current privacy protection methods, a differential privacy protection method is proposed to control user’s personal tendency and prevent sensitive information from leaking. User’s tendency is expressed as a state transition probability via the Markov decision process.Access and transition probabilities are calculated by analyzing a user’s trajectory, and a user’s access probability is converted into the weight of the Markov chain. The Markov decision process is used to analyze and quantify a user’s tendency. Thus, a transformation from a qualitative description of a user’s tendency to a quantitative representation is made by extending the differential privacy model. A privacy model uses parameters to combine the user’s propensity probability and the differential privacy budget, it can dynamically add differences and appropriate amount of noise to control user access tendency, protect user’s tendency privacy information and improve data availability. Finally, the feasibility and effectiveness of the proposed method are verified on real data.

However, differential privacy is effective to protect the offline data, but there are certain limitations in protecting online data and stream data, and the privacy protection effect will also be affected by the allocation of privacy budget parameters. Therefore, how to scientifically set acceptable privacy parameters to ensure the availability and consistency of published data which are the contents of the next research.

## Supporting information

S1 Data (ZIP)

S2 Data (ZIP)

S3 Data (ZIP)

S4 Data (ZIP)

S5 Data (ZIP)
